# Neglected Infections of Poverty in the United States of America

**DOI:** 10.1371/journal.pntd.0000256

**Published:** 2008-06-25

**Authors:** Peter J. Hotez

**Affiliations:** Department of Microbiology, Immunology, and Tropical Medicine, The George Washington University and Sabin Vaccine Institute, Washington, D.C., United States of America; London School of Hygiene & Tropical Medicine, United Kingdom

## Abstract

In the United States, there is a largely hidden burden of diseases caused by a group of chronic and debilitating parasitic, bacterial, and congenital infections known as the neglected infections of poverty. Like their neglected tropical disease counterparts in developing countries, the neglected infections of poverty in the US disproportionately affect impoverished and under-represented minority populations. The major neglected infections include the helminth infections, toxocariasis, strongyloidiasis, ascariasis, and cysticercosis; the intestinal protozoan infection trichomoniasis; some zoonotic bacterial infections, including leptospirosis; the vector-borne infections Chagas disease, leishmaniasis, trench fever, and dengue fever; and the congenital infections cytomegalovirus (CMV), toxoplasmosis, and syphilis. These diseases occur predominantly in people of color living in the Mississippi Delta and elsewhere in the American South, in disadvantaged urban areas, and in the US–Mexico borderlands, as well as in certain immigrant populations and disadvantaged white populations living in Appalachia. Preliminary disease burden estimates of the neglected infections of poverty indicate that tens of thousands, or in some cases, hundreds of thousands of poor Americans harbor these chronic infections, which represent some of the greatest health disparities in the United States. Specific policy recommendations include active surveillance (including newborn screening) to ascertain accurate population-based estimates of disease burden; epidemiological studies to determine the extent of autochthonous transmission of Chagas disease and other infections; mass or targeted treatments; vector control; and research and development for new control tools including improved diagnostics and accelerated development of a vaccine to prevent congenital CMV infection and congenital toxoplasmosis.

## Introduction

In the United States of America, the mortality rate resulting from infectious diseases has declined precipitously over the course of the twentieth century [1], and major scourges such as typhoid fever and malaria are no longer serious public health threats [2]. However, among the poorest populations living in the US there remains highly prevalent a group of serious parasitic and bacterial diseases such as Chagas disease, cysticercosis, and toxocariasis [3], which, like the neglected tropical diseases (NTDs), are characterized by their high prevalence, chronic and disabling features, and disproportionate effect on the poor [3],[4]. These infections occur outside of tropical regions of Africa, Asia, and Latin America, and I refer to them as *neglected infections of poverty*, because they not well known to the US public-health community, and they promote poverty because of their impact on child development, pregnancy outcomes, and worker productivity [5]. In this review I highlight the largely underappreciated burden of the neglected infections of poverty in the US and make policy recommendations for addressing such health disparities.

## The Distressed Regions of Poverty in the United States

**Figure 1:**
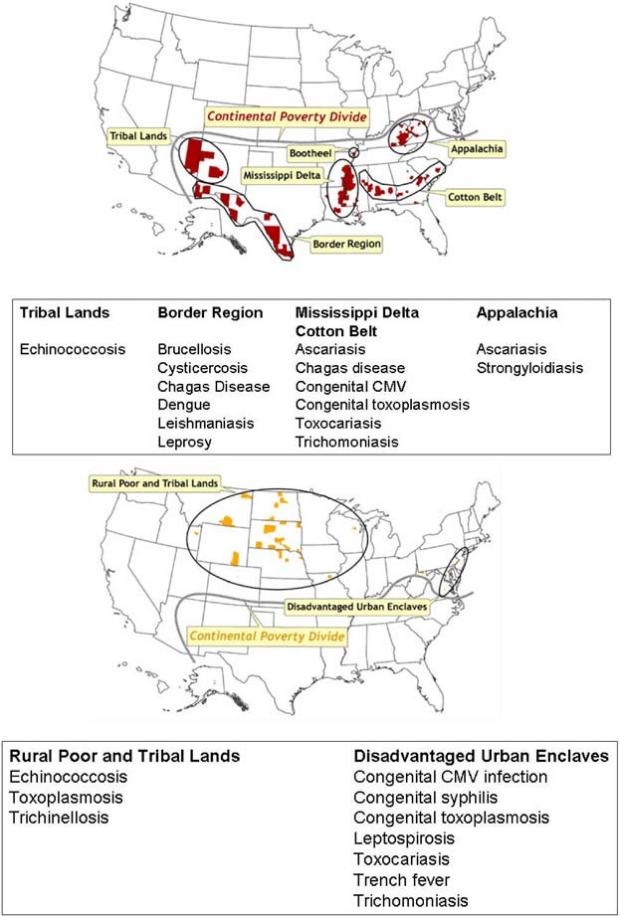
Location of Counties That Represent Spatial Clusters in Which Poverty Rates Are at Least Two Standard Deviations Higher Than the National Mean. Top: Counties south of the Continental Divide. Bottom: Counties north of the Continental Divide. From Holt [12].

Demographers and other social scientists measure poverty in a number of ways [6],[7], but since the 1960s, the US Census Bureau has used a set of income thresholds that vary by family size and composition [8],[9]. In 2006, there were 36.5 million Americans living in poverty, and the official US poverty rate was 12.3% [9],[10]. However, among under-represented minorities and children, the poverty rate is much higher, particularly in single-parent households headed by women (Table 1). Poverty in the US is not evenly distributed, but instead it is focally concentrated into several defined geographic regions, each with unique socioeconomic characteristics. Glasmeier has identified six major distressed regions of poverty: Appalachia, the Mississippi Delta, other areas of rural poverty especially in the American South, Native American tribal lands, the borderlands between the United States and Mexico, and highly racially segregated urban areas including mostly black metro areas adjacent to the Great Lakes and in the Northeast [11]. Holt has conducted a spatial analysis of the poverty in the United States at the county level and independently identified similar areas of poverty (Figure 1) [12].

**Table 1:** Selected US Census Bureau 2006 Poverty Data.

Category	Poverty Rate	Reference
Official poverty rate	12.3%	[9]
Non-Hispanic white	8.2%	[9]
Non-Hispanic black	24.3%	[9]
Hispanic	20.6%	[9]
Children under age 18 y	17.4%	[9]
Black female householder, no husband present, with children under age 18 y	43.6%	[10]
Hispanic female householder, no husband present, with children under age 18 y	42.5%	[10]

A robust dataset links poverty to both lower life expectancies from chronic diseases (especially cancer and heart disease) and increased infant and child mortality [13]–[16]. Partly on this basis, and building on an analysis of mortality by race and ethnicity in 2,077 counties or county clusters, Murray et al. divided the US population into eight groups with different epidemiologic patterns and mortality rates [14]. Among these eight “Americas” were four socioeconomically disadvantaged groups with substantially higher mortality from chronic diseases: America 4 is defined as poor whites living in Appalachia and the Mississippi Valley; America 5, Native Americans living on reservations in the West; America 7, poor blacks living in the rural South; and America 8, blacks living in high-risk urban environments [14].

Using a hybrid of these classifications it is possible to identify groups of individuals based on race, ethnicity, and socioeconomic status that are at particular risk for specific neglected infections of poverty. In this paper I review the prevalence of the major neglected diseases of poverty in the US This analysis was conducted in January 2008 using the online database PubMed [17] for 1972–2007 with the Medical Subject Headings (MSHs) “neglected diseases”, “poverty”, the specific geographic regions and racial, ethnic, and socioeconomic groups listed above [11],[12],[14], and the specific diseases listed as NTDs on the *PLoS Neglected Tropical Diseases* journal scope page [18], as well as major congenital infections associated with impaired child development including cytomegalovirus (CMV) infection, toxoplasmosis, and syphilis. I also reviewed reference lists of identified articles and hand-searched reviews. I report here either previously published estimates of the number of cases of each neglected infection, or I provide a range of estimates based on reported prevalence rates among selected communities multiplied by published estimates of the population at risk having similar socioeconomic, racial, and ethnic demographics (Table 2). For some neglected infections, particularly the soil-transmitted helminth infections, no new surveys have been reported since the 1980s. Some of the regional and national prevalence estimates were modified from a chapter in my recently published book on neglected tropical diseases [19].

**Table 2:** Estimated Prevalence of Neglected Infections of Poverty in the US.

Neglected Disease Category	Disease	Estimated Number of Cases	Major Regions or Populations at Risk	References
**Soil-transmitted helminth infections**	Ascariasis	<4 million	Appalachia, American South	[29]
	Toxocariasis	1.3–2.8 million	Inner cities, American South, Appalachia	[14],[79],[84]
	Strongyloidiasis	68,000–100,000	Appalachia, African refugees	[14],[19],[25],[35]
	Trichinellosis	16 (insufficient data)	Arctic Alaska	[149]
**Platyhelminth Infections**	Cysticercosis	41,400–169,000	US–Mexico borderlands	[19],[96],[113]
	Schistosomiasis	8,000	African refugees	[89],[90]
	Echinococcosis	Insufficient data	Tribal Lands and Arctic Alaska	—
**Protozoan Infections**	Giardiasis	2.0–2.5 million	All regions	[123],[147]
	Trichomoniasis	880,000 (black women)	American South, inner cities	[14],[66]
	Cryptosporidiosis	300,000	All regions	[123]
	Chagas disease	3,000 to >1 million	US–Mexico borderlands, American South	[11],[102],[103],[105],[109]
	Cyclosporiasis	16,624	All regions	[123]
	Congenital toxoplasmosis	≤4,000 annually	American South, inner cities, US–Mexico borderlands, Arctic Alaska	[65]
	Leishmaniasis	Insufficient data	US–Mexico borderlands	—
	Amebiasis	Insufficient data	US–Mexico borderlands	—
**Bacterial Infections**	Congenital syphilis	1,528 between 2000 and 2002	American South, inner cities	[62]
	Brucellosis	1,554	US–Mexico borderlands	[122],[123]
	Bovine tuberculosis	129 cases between 1994 and 2000	US–Mexico borderlands	[124]
	Leprosy	166	US–Mexico borderlands	[148]
	Trench fever	Insufficient data	Inner cities	—
	Leptospirosis	Insufficient data	Inner cities	—
**Viral Infections**	Dengue fever	110,000–200,000 new infections annually	US–Mexico borderlands, American South	[11],[95],[96]
	Congenital CMV	27,002 annually; 6,652 in blacks; 4,196 in Hispanics	American South, inner cities	[64]
	Human rabies	2	All regions	[149]

### Appalachia

The hilly and mountainous region known as Appalachia comprises parts of 13 states (Figure 1) [11]. Poverty and isolation is particularly severe in Central Appalachia, which includes parts of West Virginia, Eastern Kentucky and Tennessee, and the southwestern tip of Virginia [11]. The plight of the poorest people in this region, typically those working in the coal mining industry, was brought to national attention both during the early 1960s when John F. Kennedy made a presidential campaign swing through the region [20] and with the 1962 publication of Michael Harrington's book, *The Other America: Poverty in the United States* [21]. In 2000, it was estimated that 169,000 housing units in Appalachia, particularly Central Appalachia, had no indoor plumbing [11]. Almost 3% of the region overall lacks complete plumbing, although in some counties plumbing is incomplete in upwards of 25% of the housing units [11].

#### Ascariasis

The parasitic worm infection ascariasis is one of the world's most common neglected tropical diseases [4], and a leading global cause of impaired child development [22]. In very young children, high-intensity *Ascaris lumbricoides* infections also cause intestinal obstruction [22],[23]. During the 1930s, the profound poverty and inadequate sanitation in Appalachia was linked to high rates of ascariasis [24]. For instance, it was noted that among children aged 5–14 y, the prevalence in Breathitt County in Eastern Kentucky was 75%, higher than in many developing countries [24]. During the late 1970s Walzer et al. reported that approximately 14% of schoolchildren in Clay County (Eastern Kentucky) were infected with *A. lumbricoides* and almost 13% were also infected with the whipworm *Trichuris trichiura* [25], while other investigators also reported that ascariasis was still highly endemic in the region [26]–[28]. Warren previously estimated that four million people are infected with *A. lumbricoides* in the US (Table 2) [29]: however, no surveys for ascariasis have since been conducted.

#### Strongyloidiasis

Strongyloidiasis, caused by the threadworm *Strongyloides stercoralis*, is another important soil-transmitted helminth infection, associated with chronic enteritis, impaired child development, eosinophilia, and hyperinfection in immunocompromised hosts [30]–[32]. The disease is under-reported partly because of the difficulty of diagnosing the infection by fecal examination [32],[33]. A review of several studies conducted during the 1960s, 70s, and 80s and determined that the prevalence in Central Appalachia ranged from 0.4% (Charleston, West Virginia) to 4.0% (Harlan County, Kentucky, and Johnson City, Tennessee) [32]. Based on 3,271 fecal examinations in Kentucky, Walzer et al. estimated that the overall prevalence was approximately 1% [25]. A high percentage of the patients with strongyloidiasis were found to be older white males, most of whom had underlying chronic illnesses including chronic obstructive pulmonary disease [25],[30],[32],[34]. These infections may have been acquired in coal mines. Murray et al. determined that 11 million people compose the poor white Appalachians in America 4 [14], while the Centers for Disease Control and Prevention (CDC) reported that the population of rural Appalachia is approximately 6.8 million [35]. Based on Walzer's prevalence determination of 1%, I estimate there are approximately 68,000 [19] to 110,000 Appalachians infected with *S. stercoralis* (Table 2).

### Mississippi Delta and the American South

Throughout the twentieth century and continuing today, the Mississippi Delta (“the Delta,” composed predominantly of the Delta regions of Mississippi, Louisiana, Arkansas, and Tennessee, but also including the adjacent “boot-heel” of Missouri) and the areas of the former Cotton Belt in the American South, have remained among the poorest regions in the nation (Figure 1) [11]. High rural poverty rates, inadequate housing, and poor health are the hallmarks of poverty in the Delta and adjacent regions [11],[36]. More than one-half of the population is black [37], and over one-third of the total Delta black population lives in poverty, as does almost one-half of the rural black Delta population [11]. Overall, 5.8 million people live in America 7, the poor blacks of the rural American South [14].

In the first half of the twentieth century as many as 42% of black schoolchildren in the Delta had splenomegaly indicative of active malaria infection, and almost twice as many blacks died from malaria as whites [2]. The high rates of malaria among blacks were attributed to exposure to *Anopheles* mosquitoes as a result of crowding and inadequate housing located next to swampy land, and diminished host resistance because of malnourishment and overwork [2]. Throughout the American South during the early twentieth century, malaria combined with hookworm infection and pellagra to produce a generation of anemic, weak, and unproductive children and adults [2],[19],[38],[39]. By the 1960s these infections were no longer endemic in the Delta, but the health status (as measured by cancer and heart disease mortality rates and infant mortality rates) of the eight states that make up the this region still consistently ranks at the bottom among all the United States [36]. Tuberculosis rates among southern blacks are also considerably higher than whites [40]–[42]. Poverty is a major determinant but not the only one [40], as incarceration and other involuntary social forces also account for high rates of tuberculosis and some sexually transmitted infections [40], [42]–[44]. For the blacks living in the Delta and elsewhere in the American South, several parasitic and congenital infections rank among the most important neglected infections of poverty, especially in post-Katrina Louisiana.

#### Neglected infections in pre- and post-Katrina Louisiana

Despite the apparent eradication of malaria and hookworm infection from the American South [2],[19],[38],[45], other important parasitic infections remain, particularly in Louisiana. Even before Hurricane Katrina, the Delta region of Louisiana exhibited some of the highest poverty rates in the nation—in 2000, approximately 36% of blacks lived below the poverty level in this area [11]. It was previously determined that, outside of Appalachia, Louisiana exhibited some of the highest rates of ascariasis in the US [24], and during the 1970s and 1980s considerable numbers of the rural residents of Louisiana and elsewhere in the American South were infected [23], [46]–[50]. Some children exhibited parasite intensities high enough to produce acute intestinal obstruction [23],[28]. Although *A. lumbricoides* infections were highest in rural Louisiana, they were also prevalent among kindergarten children living in New Orleans [51]. In addition, during the 1970s and 1980s Louisiana children were at risk for infection with the dog roundworm, *Toxocara canis* [52], and up to 30% of rural black children, mostly in the South, were seropositive for this infection (toxocariasis will be discussed in the section on inner cities) [53]. Unfortunately, no surveys for either ascariasis or toxocariasis in Louisiana have been published since the 1980s.

Following the devastation of Hurricane Katrina in 2005, prolonged flooding combined with poverty to create conditions that could promote the emergence of additional neglected infections, including vector-borne viral diseases such as dengue fever [54]–[56] and Chagas disease [57],[58]. Chagas disease is of particular concern, because of the noted rise in domestic triatomines, especially *Triatoma sanguisuga*, which transmits the causative American trypanosome *Trypanosoma cruzi* [57],[58]. In Louisiana, almost 30% of the armadillos and 38% of the opossums are infected with *T. cruzi*, and a case of Chagas disease was recently reported in post-Katrina New Orleans [57]. Therefore, many of the requirements for autochthonous Chagas disease transmission are in place in Louisiana [58], with an established case already present. In the coming decade, global warming and increased flooding in the region could combine to promote dengue and Chagas disease epidemics among the poor in Louisiana [55].

#### The feminization of poverty

The term “feminization of poverty” refers to the observation that in the US and elsewhere women often have fewer economic resources than do men and are more likely to be heads of single-parent families [59]. Poverty is particularly feminized among black women [59]. As shown in Table 1, almost one-half of black female heads of single-parent households live below the poverty level, and black mothers are twice as likely to have premature or low birth weight infants or to have infants that die in infancy than white mothers [16]. Congenital infections, typically the result of primary cytomegalovirus (CMV) infection, toxoplasmosis, or syphilis during pregnancy, are important factors underlying these high rates of poor birth outcome. These congenital infections cause devastating long-term neurological dysfunction including cognitive impairments, intellectual retardation, and hearing and vision loss [60]–[62]. In this way, the major congenital infections are also important poverty-promoting factors causing billions of dollars in economic losses [60]. In the US, black children and their mothers bear a disproportionate congenital disease burden [63]. With respect to congenital CMV, black women exhibit a 4-fold increase in primary infection during pregnancy compared to white women, and when stratified for women between the ages of 12 and 19 there is almost a 50-fold increase [64]. Of the estimated 27,002 primary CMV infections in pregnancy in the US estimated to occur annually, 6,652 of them occur in black women (Table 2) [64]. Similarly, almost 55% of the cases of congenital syphilis occur among blacks [62], and blacks suffer from higher rates of toxoplasmosis than do whites (Table 2) [65]. In addition to primary infections during pregnancy and congenital infections, black women also exhibit an approximately 10-fold higher prevalence of trichomoniasis (13.3%) than white (1.3%) women [66]. Based on Murray's estimate that 13.3 million blacks live either in America 7 (rural South) and in America 8 (high-risk urban environments [14]), I estimate that approximately 880,000 black women in the US are infected with the protozoan parasite *Trichomonas vaginalis* (Table 2).

### Disadvantaged Urban Enclaves (Inner Cities)

High-poverty areas in American inner cities are sometimes defined as neighborhoods where more than 40% of the population is poor [67]. Jargowsky described such neighborhoods as ones that “tend to have a threatening appearance marked by dilapidated housing, vacant units with broken or boarded up windows, abandoned or burned out cars, and men ‘hanging out’ on street corners” [67]. One measure of inner city poverty used by sociologists and economists is a dissimilarity index, which measures the degree of segregation by race and income, with blacks living in the poorest neighborhoods [11]. The cities with the highest dissimilarity index are the Northeastern cities and the Midwestern cities near the Great Lakes (Figure 1) [11]. Several neglected infections are present in these and other disadvantaged urban enclaves.

#### Rat-borne and louse-borne bacterial infections

Over the last two decades, outbreaks of leptospirosis, a bacterial infection transmitted through rat urine and responsible for a serious hemorrhagic complication known as Weil's disease, have been reported among the poor living in Baltimore [68] and Detroit [69],[70]. Similarly, bartonellosis, caused by the gram-negative bacterium *Bartonella quintana*, has emerged among the homeless [71]–[73]. *B. quintana* is the cause of louse-borne trench fever, so named because it was common among soldiers living under extreme conditions in the trenches during World War I [71],[72]. Beginning in the 1990s, small outbreaks of *B. quintana* bacteremia and endocarditis was noted among the homeless living in Seattle, Washington, and elsewhere [71]–[73]. With global warming and increased flooding such rat- and louse-borne infections may increase among the homeless [55].

#### Toxocariasis

Toxocariasis is an important neglected infection of poverty among socioeconomically disadvantaged black children [53],[74],[75]. Playgrounds and sandboxes in poor urban neighborhoods are often contaminated with eggs of the dog roundworm, *Toxocara canis* [75],[76]. When children accidentally ingest these roundworms eggs the released larvae migrate through tissues to cause visceral larval migrans and eosinophilic granuloma of the liver [74],[75],[77] or ocular larva migrans [74],[78]. Another form of the disease, covert toxocariasis, has been associated with asthma [75],[79],[80], and may possibly be linked to the rise in asthma observed in inner city children [81], as well as impaired cognitive development and lower intelligence [75],[82],[83]. Based on serologic studies that measure antibody to *T. canis* antigens, the prevalence rate of toxocariasis among inner city blacks living in Connecticut cities was found to be 10% and even higher among inner city Hispanics [79]. As noted previously, the prevalence among socioeconomically disadvantaged blacks in the American South was as high as 30% [53]. In an unpublished study from the CDC it was recently estimated that approximately 21% of blacks are seropositive ([84] and Peter Schantz, personal communication), indicating exposure to the parasite. I previously estimated that approximately 500,000 blacks are seropositive for *T. canis* antibody [19]. However, based on the estimate that 13.3 million impoverished blacks live in America 7 and 8 [14] and prevalence estimates between 10% and 21%, as many as 1.3 million to 2.8 million individuals may be exposed or infected (Table 2).

### African Refugees and Other Special Immigrant Groups

Since the 1980s, the US has relocated and successfully treated populations of refugees from Southeast Asia and other developing regions with high prevalence rates of helminth infections—especially hookworm infection, filarial infections, and strongyloidiasis [85]–[87]—tuberculosis, and hepatitis B [88]. Beginning in 2000, the immigration of refugees from sub-Saharan Africa markedly increased [89], and today the US settles an estimated 70,000 refugees annually, including 25,000 refugees from Africa [90]. Notable among the refugees are the “Lost Boys and Girls of Sudan,” raised initially in poor Ethiopian refugee camps before relocating to Kenya [89]. Since 2000, almost 4,000 Lost Boys and Girls have been settled in the US. By serologic testing it was determined that almost one-half of these special immigrants are seropositive for both schistosomiasis (mostly *Schistosoma mansoni* infection) and strongyloidiasis [90]. In addition, an estimated 8,000 Somali Bantu have been relocated to the US, with up to three-fourths of them seropositive for schistosomiasis (most likely *Schistosoma haematobium* infection) and one-fourth positive for strongyloidiasis [90]. It is generally accepted that seropositivity for these two parasitic infections is a result of chronic and persistent untreated infections [89]. Therefore, of the roughly 4,000 Sudanese immigrants and 8,000 Somali immigrants there are approximately 8,000 cases of schistosomiasis and 3,000 cases of strongyloidiasis (Table 2). Accordingly, the CDC now recommends presumptive treatment for these special immigrant populations with anthelminthics [89]–[91].

### The Borderlands of Mexico

An estimated 10 million people live in the border region between the US and Mexico, many of whom are of Hispanic heritage (the majority American citizens) (Figure 1) [11]. These border communities are among the poorest in the US, and substandard or inadequate housing is common to the region [11]. Several important neglected infections of poverty occur in this setting, including vector-borne diseases, helminth infections, and other zoonoses. A related at-risk population is the estimated 750,000 to 12 million migrant farm laborers from Mexico and Central America [92].

#### Vector-borne diseases: Dengue, Chagas disease, and leishmaniasis

Poor housing without plumbing, air conditioning, or window screens is a key factor in promoting vector-borne diseases [93]. It has been estimated that this situation describes more than 30,000 border households, in addition to large numbers of mobile homes in the region [11]. Over the 20-y period between 1980 and 1999 there were 65,514 cases of dengue fever reported from the Mexico side of the border, compared to only 64 cases in the US [55],[94],[95]. An earlier assessment suggested that the higher-quality dwellings on the US side accounted for this disparity [55]; however, more recent studies indicate that dengue is under-reported in the US near the Mexican border [95]. A cross-sectional survey in Brownsville, Texas and Matamoros Tamaulipas, Mexico detected 2% and 7.3% recent infections, respectively, with evidence of past infection in 40% of Brownsville residents [95]. Risk factors and predictors of dengue among the Brownsville residents include low weekly family income, absence of air conditioning, and inadequate street drainage [95]. Assuming that 10 million people live in the US–Mexico borderlands, a 2% prevalence of recent infections [95] translates to approximately 200,000 people with recent dengue fever (Table 2). Alternatively, the Pew Hispanic Center estimates that there are 26,784,268 Mexican Americans living in the US [96]. At an overall poverty rate of 20.6% for Hispanics in the US (Table 1), there are almost six million impoverished Mexican Americans in the US. If 2% of this population suffers from a recent dengue infection, I estimate there are 110,000 recent dengue infections in the US (Table 2).

In addition to evidence for Chagas disease in post-Katrina Louisiana as described above, the US borderlands with Mexico have also emerged as an endemic region [97]–[109]. Because of concerns about the risk of new contamination of the national blood supply with *T. cruzi* [98],[100],[101],[103], with a recent estimate that between 1 in 4,655 and 1 in 25,000 US blood donors are seropositive for *T. cruzi* antibodies and presumed infected [100],[109], there is great interest in expanding current blood screening efforts [98]. In 2006, the US Food and Drug Administration approved a new commercial ELISA test for blood donation screening that utilizes parasite lysate antigens for detection of antibodies [98],[100]. Estimates of the prevalence of Chagas disease along the Mexico border and in the US vary widely. Previously, it was estimated that 50,000 to 100,000 Latin American immigrants in the US are infected [103], but more recently it was found that of 10,192 blood specimens from El Paso, Texas, of which 73% were from donors of Hispanic origin, three donors were positive [109]. With an overall prevalence of 0.03% [109] and 10 million people living in the US–Mexico borderlands [11], I estimate that approximately 3,000 people have Chagas disease in the region. Other estimates are considerably higher. Milei et al. argued that there are 370,000 *T. cruzi*–infected individuals in the US during the 1990s [105], while Hanford et al. revised these estimates to suggest that over one million Hispanics in the US have Chagas disease (with almost 270,000 in Texas alone) and that at least 150,000 Latin America–born immigrants are expected to develop clinically apparent chronic Chagas disease [102]. Congenital Chagas disease may also occur [98],[110]. Of particular concern is the possibility that *T. cruzi* transmission to humans today occurs in the US–Mexico borderlands. In South Texas and elsewhere along the US–Mexico borderlands, dogs and coyotes are seropositive and there is a domestic canine transmission cycle [97]. In addition, wood rats are common hosts, and the infection occurs among domestic cattle, horses, and sheep [102]. Infected vectors or hosts are present in 64 of the 254 counties in Texas [102], so people living in the estimated 30,000 poor-quality dwellings in the borderlands region are at high risk for transmission.

Another vector-borne neglected disease, cutaneous leishmaniasis, is transmitted by sandflies and is endemic in Mexico and Central America. Infection with *Leishmania mexicana* has been reported from South Texas, including among individuals with no travel history [111],[112]; wood rats or other rodents may also serve as reservoir hosts.

#### Cysticercosis and other zoonoses

Cysticercosis results when humans accidentally ingest eggs of the pork tapeworm, *Taenia solium*, which are shed or excreted by close household or family contacts. This condition is now a leading cause of epilepsy, seizures, and other neurological sequelae in the US–Mexico borderlands [113]–[120], accounting for approximately 10% of seizures presenting to emergency rooms in Los Angeles and, presumably, other border cities as well [116]. With an incidence rate of 8 to 10 per 100,000 per year among Hispanic populations [117],[119], I previously estimated that up to 3,500 new cases of cysticercosis occur annually [19]. In a seroprevalence study of rural Ventura County, California, it was found that 1.8% of that population have cysticercosis [113],[114]. I previously reported that there are 41,400 Hispanics in the US with cysticercosis [19], but based on the observation that 9.4 million Hispanics live in poverty in the US [96], the number of people with cysticercosis may be substantially higher. If 1.8% of this population is also infected, there may be as many as 169,000 cases of cysticercosis among Hispanics in the US (Table 2).

There are two other zoonoses of medical importance in the US–Mexico borderlands. Brucellosis is one of the most common zoonosis worldwide and a leading cause of disability [121]. Goat and cow dairy products are an important source of infection from Mexico [122], with 1,056 cases of brucellosis reported between 1993 and 2002 (although Mead et al. estimated that 1,554 cases occur annually [123]), of which almost 80% of the cases occur among individuals of Hispanic origin [122]. Between 1994 and 2000, 129 cases of bovine tuberculosis (*Mycobacterium bovis*) were reported, nearly all among patients of Hispanic origin, particularly children [124].

#### Neglected infections among migrant farm workers

Approximately 95% of the several million migrant agricultural workers in the US were born in Mexico, and almost all of them live below the poverty line [92]. They have significant health disparities, with case fatality rates more than five times the US average. In addition to very high rates of HIV, tuberculosis, and chronic diseases [92], [125]–[127], the Mexican-born migrant workers living in the US often suffer from high rates of parasitic infection, including ascariasis and hookworm infection [92], [128]–[130] (for which there is evidence of autochthonous transmission on US farms [131]), cysticercosis and Chagas disease [127],[131], and other neglected infections [92],[125].

### Tribal Lands and Arctic Native Americans

Approximately 4 million Native Americans are distributed among 500 tribes in the United States, with approximately one-fourth living on tribal lands or lands specifically designated as Native American lands (Figure 1) [11]. Almost 30% of those living on tribal lands live in poverty, where the child poverty rates are more than 40% [11].

#### Neglected infections in continental US tribal lands

Across the US, Native Americans are highly susceptible to diabetes mellitus and obesity, and almost one-third of Native Americans die before the age of 45 [11]. Up to 40% of Native Americans also live in overcrowded conditions [11], and because of this and for additional reasons of genetic susceptibility and low vaccine coverage, high rates of invasive bacterial and viral respiratory infections occur among Native Americans, especially the Navajo and Apache [132]–[135]. On some reservations up to one in five homes lack complete in-house plumbing, a rate that is 20 times the national average [11]. In this setting, certain neglected infections of poverty are common. Over the last twenty years in the American Southwest, trachoma has been common among the Navajo [136],[137], while cystic echinococcosis has been endemic among the Navajo, Zuni, and Santo Domingo Indians because of an enzootic dog–sheep cycle on tribal lands and elsewhere in the region [138]–[140].

#### Neglected infections among the Inuit

Because of their dietary reliance on meat from sea mammals and polar bear the Inuit living in Alaska and the Canadian Arctic are at risk of food-borne parasitic diseases, including echinococcosis, toxoplasmosis and congenital toxoplasmosis, and trichinellosis [19]. Cystic echinococcosis in the Arctic is due to an enzootic cycle involving moose, reindeer, and elk [141], while trichinellosis caused by *Trichinella spiralis nativa* is prevalent because of high rates of infection among walruses and polar bear [142]. Toxoplasmosis and congenital toxoplasmosis are also extremely common among the Inuit, and are due to consumption of infected seal and caribou meat [143].

### Other Regions

The most diagnosed parasitic in the infection in the US is giardiasis [144],[145], with as many as 2.0–2.5 million cases occurring annually [123],[146]. The greatest number of cases occurs between June and October and among children aged 1–4 and 5–9 y and adults aged 35–39 y [144]. An estimated 300,000 cases of cryptosporidiosis also occur annually [123], and this infection has emerged as a leading cause of recreational water outbreaks of diarrhea in the US and among patients with HIV/AIDS [147]. A 10-fold increase in cryptosporidiosis transmission occurs during the summer and early fall [147]. Although both giardiasis and cryptosporidiosis are common, there is no evidence to suggest that they disproportionately affect poor and under-represented minority populations. In contrast, the intestinal protozoan disease amebiasis does disproportionately affect the poor, but no US prevalence data are available for this disease. Among the notifiable neglected infections of poverty there were 166 cases of leprosy (with most of the cases in Texas, California, New York and Louisiana [148]), 16 cases of trichinellosis, and two cases of human rabies reported in 2005 (Table 2) [149].

## Policy Recommendations

Based on my estimates of prevalence (Table 2) and other health and socioeconomic impacts, the most important neglected helminth infections of poverty in the US are the helminth diseases toxocariasis (inner cities and the American South), ascariasis (Appalachia and the American South), strongyloidiasis (Appalachia), and cysticercosis (US–Mexico borderlands). Among the important vector-borne neglected infections are dengue and Chagas disease in the US–Mexico borderlands and in post-Katrina Louisiana. Congenital infections such as congenital CMV and congenital syphilis stand out as health disparities in inner cities and the American South. Trench fever and leptospirosis are important among the homeless and other disadvantaged urban populations.

Among the common features of these neglected infections are (1) their highly disproportionate health impact on people of color and people living in poverty; (2) their chronic, largely insidious, and disabling features; and (3) their ability to promote poverty because of their impact on child development, pregnancy outcome, and productive capacity. It is important to note that, while some of these neglected infections occur exclusively among recent immigrant populations, most do not. Instead, poverty is the single most important determinant. Control of these neglected infections needs to be prioritized by policy makers and public-health experts because it is both a highly cost-effective mechanism for lifting disadvantaged populations out of poverty and consistent with our shared American values of equity and equality [150]. The World Health Organization also recognizes that control of neglected diseases represents a fundamental human right [151].

An important obstacle to the control or elimination of the neglected infections of poverty in the US is the absence of reliable population-based estimates of prevalence and disease burden data about these conditions [3],[19]. These neglected infections are underdiagnosed and most are not reportable to the CDC. The estimates I provide here are preliminary and based on very few active surveillance studies, including some obtained by analyses of sera collected from National Health and Examination Surveys. For some of the neglected infections of poverty, seropositivity may be equated with active infection [89],[90],[95],[109],[113], whereas for others it may reflect both current and past infections [53],[74]. For infections such as Chagas disease estimates reported here vary widely. We also lack a system for the national collection of fecal samples for intestinal parasitic infections. Expanded measures are urgently needed to implement active surveillance and obtain population-based estimates of the neglected infections (Table 3). An added measure would be to expand newborn screening for toxoplasmosis [3],[152], and possibly congenital Chagas disease. Screening for congenital toxoplamosis would also likely benefit persons of all socioeconomic circumstances [61]. Such efforts would create opportunities to determine the extent and true disease burden of these neglected infections.

**Table 3:** Priority Needs for Enhanced Surveillance, Treatment, and Prevention Efforts for the High Priority Neglected Infections of Poverty.

Disease Category	Disease	Expanded Active Surveillance and Treatment	Newborn Screening and Treatment	Epidemiological Transmission Studies	New Diagnostics	New Drugs	New Vaccines
**Helminth Infections**	Ascariasis	+		+			
	Toxocariasis	+		+	+		
	Strongyloidiasis	+		+	+		
	Cysticercosis	+		+	+	+	
**Protozoan Infections**	Giardiasis	+					
	Cryptosporidiosis	+		+		+	
	Trichomoniasis	+					
	Chagas disease	+	+	+	+	+	+
	Leishmaniasis	+		+	+	+	
	Congenital toxoplasmosis	+	+	+	+	+	+
**Bacterial Infections**	Congenital syphilis		+	+			
	Brucellosis	+		+			
	Bovine tuberculosis	+		+			
	Trench fever	+		+			
	Leptospirosis	+		+			
**Viral Infections**	Dengue fever	+		+		+	+
	Congenital CMV	+	+	+		+	+

There is also an urgent need to better define the transmission dynamics of some of the neglected diseases (Table 3). For Chagas disease, and to some extent, dengue and leishmaniasis, the full extent of authochthonous transmission in Louisiana and the US–Mexico borderlands is poorly understood. A full appreciation of Chagas disease transmission mechanisms would include molecular genotyping of the parasite to determine whether different strains or demes are endemic, and a complete characterization of the different vectors and animal reservoir hosts. Similarly, the extent of autochthonous cysticercosis transmission in the US is largely unstudied, as it is for many of the bacterial zoonoses including urban foci of leptospirosis and trench fever. For toxocariasis, the contribution of feral versus domesticated animal reservoirs to transmission is also not well understood.

Following enhanced surveillance and improved understanding of transmission dynamics, there are several opportunities to treat or prevent neglected infections of poverty in the US using existing drugs or other control tools (Table 3). Through either population-based drug administration or case identification and treatment, the soil-transmitted helminths could be controlled by administration of albendazole and ivermectin [22], while expanded use of praziquantel would treat schistosomiasis among selected immigrant populations [90] and prevent transmission of *T. solium* eggs and possibly reduce the incidence of cysticercosis [153]. Metronidazole and tinidazole are available for the treatment of trichomoniasis and giardiasis [154],[155], and nitazoxanide is available for cryptosporidiosis and giardiasis [144],[156]. Pyrimethamine plus sulfadiazine is used for the treatment of toxoplasmosis, and the optimal length of treatment and its impact on child development and neurological sequelae need to be determined [61]. Antibiotics are available for the treatment of leptospirosis and other bacterial zoonoses [157]. An important role also exists for veterinary public health interventions to prevent zoonotic transmission to humans, possibly including the mass treatment of *Toxocara*-infected dogs, *Toxoplasma*-infected cats, and other measures [158]. The control of almost all of the neglected infections of poverty would also benefit from improvements in environmental sanitation, piped clean water, and improvements in housing in some of the poorest endemic areas. For Chagas disease, dengue, and leishmaniasis, consideration of expanded vector control approaches is warranted [55],[159].

Development of new control and prevention tools is needed (Table 3). Currently, the serologic-based diagnostic tests for most of the parasitic infections rely on extracts or crude preparations of parasite antigens and would benefit from the development of improved and widely available diagnostic kits that utilize standardized and purified recombinant antigens. For Chagas disease there is a particularly urgent need for rapid diagnostic tests and polymerase chain reaction-based assays for detection of acute and congenital infections. Furthermore, no drugs adequately and reliably treat Chagas disease [160], dengue [161], or congenital CMV infection [162]. Although vaccines for dengue [163] and CMV infection [164] are under development, progress has been slow because of inadequate resources and commercial incentives [5]. A pediatric dengue vaccine initiative was recently established through support by the Gates Foundation [163]. For CMV infection, both a live attenuated vaccine and a recombinant vaccine have been developed [164], but clinical testing in pregnant women to determine the impact of these vaccines on vertical transmission has been severely lagging because of inadequate support—a tragedy, given that more than 10,000 congenital CMV infections occur among infants of color annually [64].

In 2006, the annual budget of the National Institute of Allergy and Infectious Diseases (NIAID) was $4.4 billion, with approximately $1.6 billion of this amount spent on biodefense [165]. Of the selected disease-specific areas targeted for funding by the NIAID in their published annual report, none specifically mentions a neglected infection of poverty [165]. A consequence of this lack of targeted funding for neglected diseases is that the development of critically needed new tools for these conditions has lagged behind those for biodefense. The Global Forum on Health Research has coined the term “the 10/90 gap” to describe how only 10% of resources are devoted to 90% of the global burden of disease, i.e., that represented by disease disproportionately occurring in developing countries [166]. The absence of development of new tools for neglected infections of poverty, such as those outlined above, highlights a unique American 10/90 gap for poor people and people of color in the US.
